# Radionuclides transform chemotherapeutics into phototherapeutics for precise treatment of disseminated cancer

**DOI:** 10.1038/s41467-017-02758-9

**Published:** 2018-01-18

**Authors:** Nalinikanth Kotagiri, Matthew L. Cooper, Michael Rettig, Christopher Egbulefu, Julie Prior, Grace Cui, Partha Karmakar, Mingzhou Zhou, Xiaoxia Yang, Gail Sudlow, Lynne Marsala, Chantiya Chanswangphuwana, Lan Lu, LeMoyne Habimana-Griffin, Monica Shokeen, Xinming Xu, Katherine Weilbaecher, Michael Tomasson, Gregory Lanza, John F. DiPersio, Samuel Achilefu

**Affiliations:** 1 Department of Radiology, Washington University School of Medicine, St. Louis, MO 63110 USA; 2 Department of Medicine, Washington University School of Medicine, St. Louis, MO 63110 USA; 3 Department of Biochemistry and Molecular Biophysics, Washington University School of Medicine, St. Louis, MO 63110 USA; 4 Department of Biomedical Engineering, Washington University, St. Louis, MO 63105 USA; 5 Present Address: James L Winkle College of Pharmacy, University of Cincinnati, Cincinnati, OH 45267 USA

## Abstract

Most cancer patients succumb to disseminated disease because conventional systemic therapies lack spatiotemporal control of their toxic effects in vivo, particularly in a complicated milieu such as bone marrow where progenitor stem cells reside. Here, we demonstrate the treatment of disseminated cancer by photoactivatable drugs using radiopharmaceuticals. An orthogonal-targeting strategy and a contact-facilitated nanomicelle technology enabled highly selective delivery and co-localization of titanocene and radiolabelled fluorodeoxyglucose in disseminated multiple myeloma cells. Selective ablation of the cancer cells was achieved without significant off-target toxicity to the resident stem cells. Genomic, proteomic and multimodal imaging analyses revealed that the downregulation of CD49d, one of the dimeric protein targets of the nanomicelles, caused therapy resistance in small clusters of cancer cells. Similar treatment of a highly metastatic breast cancer model using human serum albumin-titanocene formulation significantly inhibited cancer growth. This strategy expands the use of phototherapy for treating previously inaccessible metastatic disease.

## Abstract (web summary)

Most of the systemic cancer therapies lack spatiotemporal control. Here, the authors show targeted activation of a light-sensitive drug by radiopharmaceuticals in disseminated cancer cells as potential in vivo treatment of metastatic diseases with reduced off-target toxicity.

## Introduction

Most deadly cancers are associated with metastatic spread^1^, requiring systemic treatment strategies with chemotherapeutic drugs and radiation therapy. Second generation systemic therapies rely on targeting precise molecular signatures of cancer or invoke immune responses against certain epitopes specific to cancer. While immensely promising, on-going clinical trials indicate that these strategies are often associated with life-threatening on-target, off-tumour toxicities^2^. For many cancers, bone marrow is invariably involved as the point of origin or a distant metastatic niche^3^. The microenvironment of the bone marrow is laden with hematopoietic stem cells and progenitors, making it a highly challenging niche for selective cancer cell killing and a difficult terrain for emerging systemic therapeutics. Moreover, in advanced stages of disseminated cancers, patients often present with extremely low total lymphocyte counts. As such, they are stratified as severely immunocompromised and carry the risk of poor prognosis and low overall survival rates^4–7^. These patients are typically unsuitable candidates for existing systemic therapies and emerging immunotherapies. Photodynamic therapy or phototherapy (PT) can offer high spatiotemporal precision and control of tumour killing through a combination of direct cytotoxicity, immune-stimulatory, and antiangiogenic mechanisms^8^. Therefore, PT could serve as an effective therapeutic platform and a viable option for disseminated cancers, offering an alternative treatment for the chemotherapy-refractory disease. However, the limited penetration of external light has confined PT to the treatment of surface accessible lesions. In addition, a priori knowledge of tumour location is a prerequisite for initiating PT, which often is indeterminate in the case of disseminated tumours.

An alternative approach that delivers light or stimulate light-sensitive drugs within tissues and inside cells in vivo could facilitate the treatment of PT-inaccessible systemic and metastatic cancers. Clinically relevant radiopharmaceuticals are reliable sources of Cerenkov radiation (CR) for cancer imaging^9^. A decaying radionuclide could excite materials through, including the direct interaction of electron and positron emission with matter, particularly metals; the emission of ultraviolet-blue light emitted by beta (β) particles, known as CR, to generate cytotoxic reactive oxygen species (ROS); chemiluminescent reaction when ambient ionizing radiation excites bulk water; and emission of *γ* photons after the annihilation event. For simplicity, we group all these effects as Cerenkov radiation-induced therapy (CRIT). Therefore, a critical component of the study is to efficiently harvest the diverse potential effects of radionuclides to stimulate spatiotemporal cell death in the presence of photosensitizers. Many drugs possess photoactive properties, but the absence of a depth-independent photoelectronic energy source has confined their use as chemotherapeutics, preventing therapy enhancement through a complementary phototherapeutic effect.

In this study, we hypothesize that CR-mediated conversion of light-sensitive drugs to phototherapeutic agents will induce cell death through pathways distinct from the ground state drug (chemotoxicity) and in a highly selective fashion for the treatment of diverse cancer phenotypes. Using multiple myeloma (MM) and metastatic breast cancer models in mice, we demonstrate that incorporating unmodified and pristine hydrophobic light-sensitive drugs in tumour-targeted lipid nanomicelles or human serum albumin (HSA) nanoparticles, selectively deliver the agents in disseminated cancer cells. Subsequent in vivo administration of a radiopharmaceutical for CRIT inhibits the proliferation of disseminated multiple myeloma and aggressive metastatic breast cancer cells in mice. Our treatment strategy transforms chemotherapeutics to spatiotemporally photoactivatable drugs using clinically relevant radiopharmaceuticals and expands the use of phototherapy for treating previously inaccessible metastatic disease.

## Results

### Contact-facilitated drug delivery via VLA-4-targeted nanomicelles

**Fig. 1:**
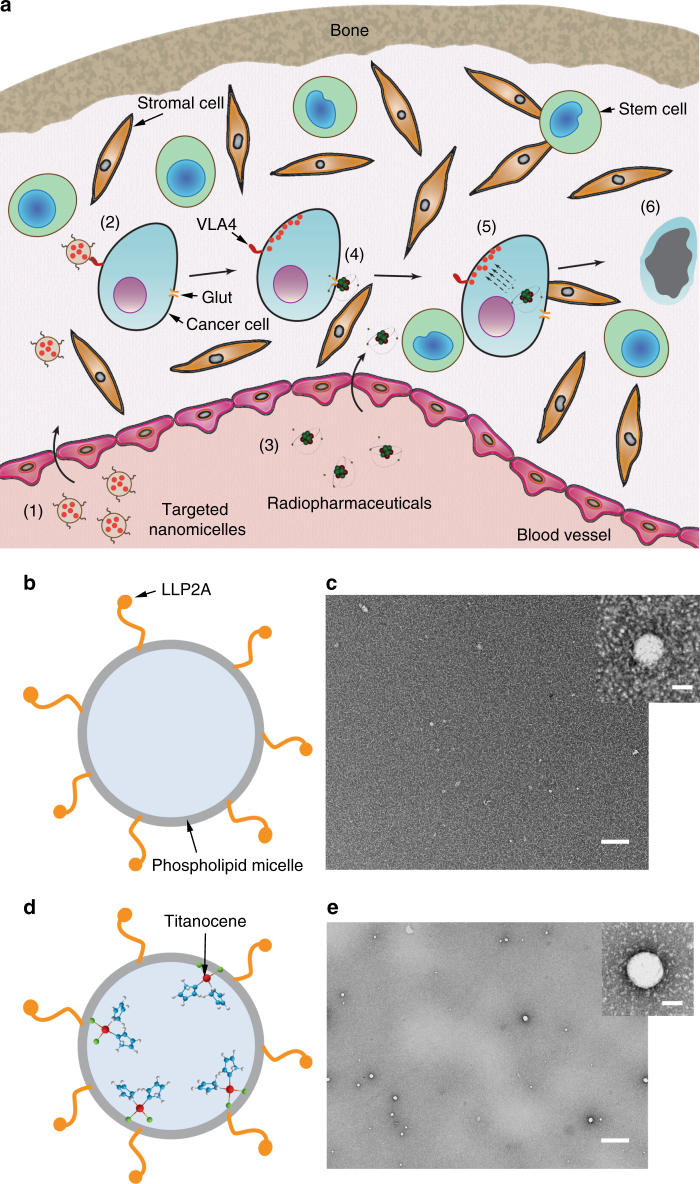
Orthogonal cancer targeting strategy using nanomicelles. **a** Schematic of the process of photoactivation of Titanocene in disseminated cancer cells in the bone marrow microenvironment. The various phases are numbered: 1. Administration of targeted NM-TC; 2. The targeted NM enter the bone marrow from the vasculature and bind to α4β1 receptor on the cancer cells and subsequently deliver the drug to the cell; 3. Administration of radiopharmaceuticals (^18^FDG), which is typically 1.5–2 h after phase (1); 4. ^18^FDG enters the cancer cells through the overexpressed Glut transporters on cancer cells; 5. Once the drug and radiopharmaceutical are co-localized in the cancer cells, the former is photoactivated by the latter through CR leading to cell death (6). Notice that since the other vital cells in the bone marrow, such as stem cells and stromal cells, do not express the combination of α4β1 and glut receptors essential for the treatment to work, they would largely remain unaffected causing minimal off-target toxicity. **b** Schematic of phospholipid NM with VLA-4 homing ligands. **c** TEM image of micelles alone. Scale bar, 100 nm. Inset: single micelle. Scale bar, 10 nm. **d** Schematic of phospholipid NM encapsulating TC with VLA-4 homing ligands. **e** TEM image of micelle incorporated with TC in the membrane. Scale bar, 100 nm. Inset: single NM-TC. Scale bar, 10 nm

Targeted delivery of a radionuclide and a drug is necessary to enable co-localization in the same or adjacent cell for subsequent activation and therapy. Once internalized by a target cell, the radionuclide which essentially behaves as a point source of photoelectronic energy, can excite or stimulate photoactive materials in its vicinity (Fig. 1a). Previous studies have demonstrated the modularity afforded by this approach in treating cancer cells^10^ or subcutaneous solid tumours or mice using different radionuclides and photosensitizer combinations^11,12^. Unlike subcutaneous solid tumour xenografts, which do not recapitulate the physiopathology of human cancer and are treatable by conventional PT, most disseminated tumour models present a different set of challenges because they are embedded in the complex and protective microenvironment of the bone marrow^13^. As a result, it would require more effective targeting and delivery strategies to maximize cell death. We selected multiple myeloma (MM), an incurable plasma cell dyscrasia that predominantly affects the bone marrow, spleen, and bones as the representative orthotopic disseminated tumour model (Fig. 1a)^14^. We also used PyMT-BO1 cancer cell line derived from transgenic PyMT cancer cells as a highly aggressive metastatic breast cancer model^15^ (see below).

Titanocene (TC) was used in this study as the photosensitizer for several reasons, including its UV light excitability and responsiveness to low radiance of CR^11^; biodegradability with significantly low cellular footprint post therapy; ease of human translation due to its safety profile in phase 2 clinical trials^16^; and small size and lipophilicity, allowing integration into lipid-based vehicles and incorporation into cell membranes post targeting. In addition to harvesting CR luminescence, the metal centre can also interact with radiation particles to further stress cells. However, two fundamental challenges to TC and similar photoactive drugs, transvascular delivery to tumour cells and cellular localization, have remained unaddressed in the context of CRIT. In our previous study, we used transferrin (Tf) to deliver TC to tumour cells^11^. Tf has only two binding pockets for TC^17,18^. In the docking process, the cyclopentadienyl (Cp) ligands of TC can be displaced, leaving the Ti(IV) ion alone as the predominant component that binds to the pockets^17^. Because both photoactivation of Ti(IV) ion and oxidation of Cp ligand to peroxyl radical contribute to the cytotoxicity of TC, Tf-mediated transport of TC would potentially lower the therapeutic efficacy of Tf-TC.

ROS-mediated damage to lipid membranes is a primary mode of action in PT^19^. Given the short half-lives and small diffusion distance of some ROS, the mode of delivery of the drug to the target cell and its proximity to the cell membrane are important considerations for effective therapy. There is also growing evidence that therapeutic efficacy of PT can be enhanced by selective delivery of hydrophobic photoactive drugs to the plasma membrane compared to receptor-mediated endocytotic uptake^20^. The contact-facilitated delivery of drugs to the plasma membrane by lipid vehicles serves this purpose efficiently. Although liposomal formulations can deliver drugs to cells through this mechanism, conventional liposomes have an average diameter of 100 nm (for unilamellar vesicles) and 0.5–5 μm (for multilamellar vesicles)^21^, which exceeds the physiologic upper limit of 60 nm pore size for transvascular transport of macromolecules to flow across capillary walls of bone marrow^22^. To deliver pristine TC to the plasma membrane of MM cells, we used nanoscale unilamellar phospholipid micelles, also known as nanomicelles (NM), as a carrier vehicle. The NM have an average diameter of ≤15 nm, which is ideal for targeting the bone marrow interstitial space^23^. The upregulation of a key adhesion molecule, VLA-4 (α_4_β_1_ integrin), in MM provides an attractive target for precision imaging and therapy^24,25^. Human MM1.S cell line is widely used to study MM in rodents. Screening of the MM human cell line, MM1.S, using anti-CD49d (α4) and CD29 (β1) antibodies showed a ≥95% expression level of VLA-4 (Supplementary Figure 1). We loaded the NM with LLP2A (Supplementary Figure 2a), a small molecule peptidomimetic that binds VLA-4 with an exceptionally high affinity (IC50 = 2 pM)^26^. LLP2A was synthesized on a solid support, followed by conjugation to phospholipids (DSPE) engrafted with polyethylene glycol (PEG) chains to improve circulation in blood (see “Materials and Methods' section for details; Supplementary Figure 2b, c). The NM were generated as a microfluidized suspension containing LLP2A-PEG-DSPE and TC. Control NM that excluded the homing ligand LLP2A or TC were also prepared (Fig. 1b, c). An average size distribution of NM with and without TC was 14.7 ± 2 nm and 11.9 ± 0.5 nm, respectively, with an average polydispersity index of 0.2 (Table 1).

**Table 1:** Size distribution of the nanomicelles

Sample	Hydrodynamic diameter (nm)	Polydispersity index	Zeta potential (mV)
Nanomicelle + LLP2A	11.9 ± 0.5	0.217	−0.81
TC nanomicelle + LLP2A	14.7 ± 2	0.241	2.12

We successfully loaded 0.19 mg mL^−1^ of TC in the NM (Table 2). Based on the full-width half-maximum of the NM size distribution (about 15 nm), the volume of NM, and the net concentration of TC per volume of NM using inductively coupled plasma optical emission spectrometry, we determined the average number of TC per NM as 3 (range, 2–5). The incorporation of TC in the lipid layer was confirmed by electron microscopy (Fig. 1d, e). The metallic titanium (Ti) centre in TC rendered the vesicles electron dense in contrast to the control vesicles without TC. Upon addition into the NM, TC incorporated in the interface between the lipid and the hydrophilic layers, as evidenced by electron microscopy (Fig. 1d, e). Probably, the hydrolysis of TC dichloride to the dihydroxyl derivative in aqueous medium^17^ created an amphiphilic structure, favouring the orientation of the two cyclopentadienyl and dihydroxyl moieties toward the hydrophobic core and the outer hydrophilic segment, respectively. Incorporation of LLP2A did not destabilize the NM and the presence of unnatural amino acids conferred protease resistance and high plasma stability on the nanosystem^26^.

**Table 2:** Metal (Ti) and TC content in nanomicelles and HSA

Sample	Average Ti content (μg/20 μL)	Average TC content (mg/mL)
TC nanomicelle	0.52	0.192
TC-HSA	21.65	5.613

### In vivo pharmacokinetics of VLA-4-targeted nanomicelles

**Fig. 2:**
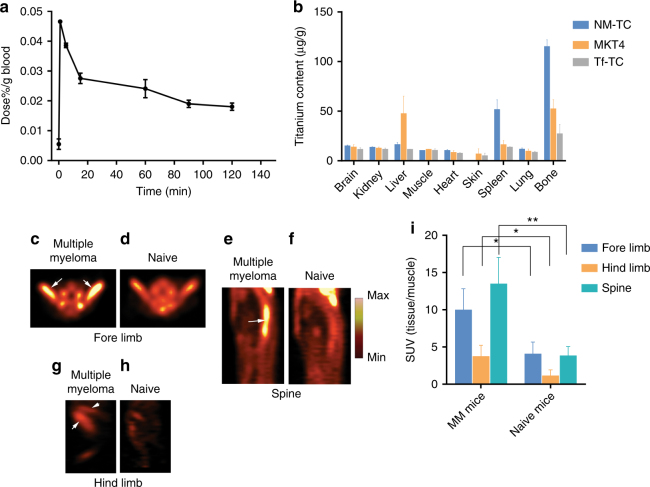
Monitoring nanomicelles biodistribution and spread of multiple myeloma in vivo. **a** Pharmacokinetics of NM-TC in rats using coupled plasma optical emission spectrometry. Half-life is 123.4 min. **b** Comparison of biodistribution in mice of targeted NM-TC and pristine TC in vivo showing highest uptake and retention in bones and spleen, characteristic of multiple myeloma, 2 h post injection. ^18^FDG-PET images showing increased uptake of ^18^FDG in mouse forelimbs, spine, and hind limbs of mice with multiple myeloma (**c**,** e**,** g**) compared to naive mice (**d**, **f**,** h**,** i**). Comparison of standard uptake values (SUV) of ^18^FDG in multiple myeloma vs. naive mice in various bones. Values are means ± s.e.m. *P < 0.05, **P < 0.01. *n* = 5 mice for each of the pharmacokinetics study in rats; and biodistribution study in mice

In vivo pharmacokinetic (PK) profile of the NM-TC was studied in naive rats. A plasma half-life of 123 min was obtained after systemic administration (Fig. 2a). We performed the PK in rats instead of mice to obtain sufficient blood sample for serial measurements of TC concentration in the same animal. Otherwise, the small volume of blood in mice would require us to pool samples from different mice, masking inter-specimen variability. Although the PK value in mice are expected to be shorter than rats, the information allowed us to estimate half-life of TC in rodent blood. The NM in circulation remained intact in vivo, until cleared or destroyed. However, the micelles have a limited half-life and must reach their target early before elimination.

### VLA-4-targeted nanomicelles delivers TC to MM-avid organs

The selectivity of LLP2A to MM cells and the serum stability of the NM in delivering the TC was determined by in vivo biodistribution analysis. Using inductively coupled plasma optical emission spectrometry, we determined the Ti metal content ex vivo in organ samples from an orthotopic disseminated MM1.S/SCID model. We compared the biodistribution of NM-TC, Tf-TC and MKT4, a water soluble analogue of TC that was used in phase 1 and phase 2 clinical trials^16,27^ at 90 min post injection. The choice of 90 min time point is based on rat PK data, which showed a *t*_1/2_ of 123 min in rats but the rate of clearance after 90 min approached stasis, probably representing the contribution of an intraversation process of drug from tissues to blood (Fig. 2a). Although this time point is expected to be shorter in mice, we chose 90 min for the mouse study to ensure that the blood concentration of TC is sufficiently low, to prevent potential systemic toxicity, but not too late when the amount in tumour tissue is small. In mice administered with NM-TC, the highest Ti concentrations were found in skeletal tissue and spleen, which typically house MM cells, with relative values of 115 ± 7 and 52 ± 9.5 μg g^−1^, respectively (Fig. 2b). In comparison, the uptake of MKT4 was lower in tumour sites, with values of 53 ± 9 and 16 ± 4 μg g^−1^, for skeletal tissue and spleen, respectively (Fig. 2b). Similarly, the accumulation of TC in these tissues for mice treated with Tf-TC was only 27.5 ± 6 and 14 ± 1 μg g^−1^, respectively. These results demonstrate the advantage of using NM to deliver TC to MM target organs. Previous studies have suggested that the cylopentadienyl rings in TC, which assists in stabilizing the Ti(IV) ion in a monomeric form, are lost in MKT4 and Tf-TC^17,18^. Thus, sequestration of TC in the hydrophobic region of NM may help stabilize the drug and minimize rapid loss from target tissues.

### CRIT inhibits tumour growth in disseminated MM mouse model

We used an FDA approved and clinically employed radiopharmaceutical, ^18^FDG (*t*_1/2_ = 109.8 min), as a source of photoelectronic energy^28^. The radiopharmaceutical, which is currently the gold standard for clinical imaging of MM^29,30^, targets metabolically active tumours via the glucose transporter (GLUT1) protein. By using an orthogonal-targeting GLUT1 and VLA-4 strategy to, respectively, deliver the ^18^FDG and NM-TC to the MM cells, we aimed to minimize the potential saturation or depletion of the targeted receptors. In healthy subjects, ^18^FDG uptake is low in the bone marrow and spleen, but significantly higher in malignancy, inflammation or after administration of hematopoietic growth factors^31^. Using small animal positron emission tomography (PET) of MM in mice, we found more than twofold uptake of ^18^FDG in bones compared to naive mice (Fig. 2c–i).

**Fig. 3:**
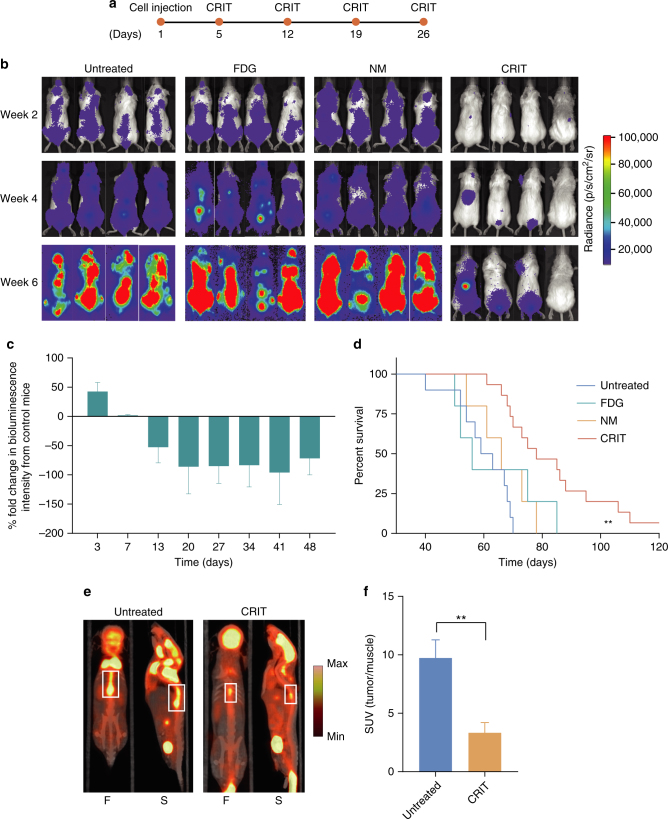
Response of multiple myeloma to CRIT. **a** Timeline of treatment. **b** Bioluminescence imaging of representative multiple myeloma-bearing mice in different treatment groups—untreated, ^18^FDG, NM controls and CRIT. All images are dorsal images and on the same scale. The images of control groups appear saturated on week 6 in comparison to CRIT. **c** Change in bioluminescence intensity as a result of treatment compared to untreated control. The intensity consistently remains lower than untreated controls during the treatment and beyond. **d** Comparison of survival of different treatment groups showing a twofold increase in survival in treated mice compared to control groups. **P < 0.01. **e** ^18^FDG-PET images of MM mice before and after treatment showing lower tumour burden in the latter. F: frontal view, S: sagittal view. Boxes denote tumour region. **f** SUV values of the treatment group were lower than untreated controls. **P < 0.01. *n* = 15 mice for CRIT, *n* = 10 mice for untreated control and *n* = 5 mice for NM-TC alone and ^18^FDG alone treated mice

The performance of CRIT in a disseminated MM1.S/ SCID mouse model was tested. Based on the biodistribution data, sequential tail vein injections of NM and then ^18^FDG were spaced 90 min apart to activate TC in tumours. Treatment was repeated four times at an interval of 1 week, and the disease progression was monitored weekly by bioluminescence imaging (BLI; Fig. 3a). A week interval was chosen for treatment for several reasons that include the need to allow the mice to fully recover from the treatment; account for full decay cycle of ^18^FDG; consider logistical reasons such as tail vein recovery; and allow sufficient time for imaging time points between treatment sessions. In the control groups consisting of untreated mice or those treated with either NM or ^18^FDG alone (Fig. 3b, c), we observed an exponential increase in the BLI signal over several weeks, demonstrating the systemic progression of the disease and indicating the primary involvement of the spleen and skeletal tissues. In contrast, mice treated with NM-TC and ^18^FDG showed a conspicuous decrease in the disease progression, suggesting the effective targeting and response of MM1.S to CRIT. Survival studies revealed a significant advantage of the CRIT over the control groups with 50% surviving up to about 90 days compared to about 62 days for the control groups (Fig. 3d). Correlative ^18^FDG-PET imaging confirmed the lower tumour burden in CRIT-treated mice compared to the control groups (Fig. 3e, f). The mice were killed after they developed hind limb paralysis resulting from spinal cord and spinal vertebral involvement. The treated mice eventually succumbed to cancer due to the remnant MM cells that could not be completely eradicated by CRIT.

### CRIT selects for α4-deficient multiple myeloma cells in vivo

**Fig. 4:**
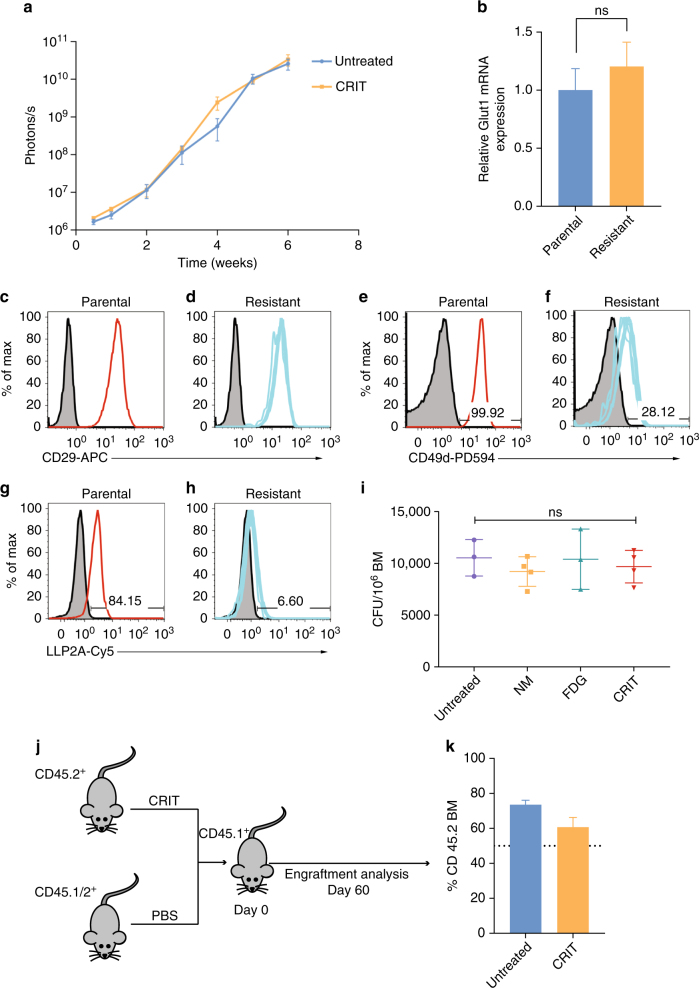
CRIT selects for CD49d cells in MM model. **a** Bioluminescence intensity plot showing resistant nature of MM cells extracted from treated cohort (MM1^CRIT-RES^) upon rechallenging with CRIT in fresh mice. **b** No difference in GLUT1 mRNA expression was observed between parental MM1.S cells and resistant MM1.S^CRIT-RES^ cells as assessed by qRT-PCR. ns not significant. **c**–**h** No difference in expression of CD29 was observed between MM1.S cells (**c**) or MM1.S^CRIT-RES^ cells (**d**) following treatment with CRIT in vivo. MM1.S stopped responding to CRIT by downregulating expression of VLA-4 subunit CD49d (Resistance = 28.12% CD49d+) (**f**) relative to parental cells injected into mice at the beginning of the experiment (parental = 99.92% CD49d+) (**e**), resulting in reduced binding of the VLA-4-targeting ligand LLP2A on resistant cells (**h**) (LLP2A+ = 6.6%) compared to parental MM1.S (**g**) (LLP2A+= 84.15%). **i** No significant difference in colony-forming units of progenitor stem cells was observed between untreated, control and treated mice. **j** To determine if CRIT reduced engraftment of haematologic cells in vivo, we assessed BM repopulation following CRIT treatment. Bone marrow from treated mice or PBS-treated controls were mixed with congenic B6.CD45.1/2 at a ratio of 1:1 before infusion of 1 × 10^6^ total BM cells into lethally irradiated (TBI 1100 cGy) B6.CD45.1 recipients. **k** Percentage of cells derived from treated donor BM (CD45.2) were calculated as a percentage of total donor BM (CD45.2 + CD45.1/2). BM from CRIT-treated mice effectively repopulated recipients (*n* = 5 per group)

Residual cancer cells that escaped treatment appeared focal and confined at random sites within the major bones, particularly the vertebrae (Fig. 3b, e). These localized cancer cells continued to grow, albeit at a slow rate. The surviving cancer cells were subsequently extracted from the mice and reintroduced into a fresh group of naive SCID mice to determine response to when treated with CRIT. However, BLI (Fig. 4a) and ^18^FDG-PET did not show noticeable differences between the treated and untreated groups, suggesting the cells were resistant to CRIT. These CRIT-resistant MM1.S (MM1.S^CRIT-RES^) cells were harvested and analysed for the expression levels of GLUT1, α4 and β1 integrins to determine whether uptake of ^18^FDG by GLUT1 or α4β1 binding of the NM were compromised. GLUT1 mRNA (Fig. 4b) or β1 cell surface expression (Fig. 4c, d) analyses did not demonstrate significant difference between the parental MM1.S and the MM1.S^CRIT-RES^ cells. However, the MM1.S^CRIT-RES^ cells expressed lower cell surface α4 than parental MM1.S cells (Fig. 4e, f). Flow cytometry analysis demonstrated that LLP2A-Cy5, which selectively binds VLA-4 with high affinity^32^, did not internalize in the MM1.S^CRIT-RES^ cells compared to the parental MM1.S cells (Fig. 4g, h). These results suggest that the MM1.S^CRIT-RES^ cells had downregulated the expression of α4 (CD49d), possibly impairing the binding of the LLP2A functionalized NM to some MM cells. Unlike in vitro studies where static incubation of nanoparticles can abrogate specific binding of receptor-targeted materials, the in vivo dynamics and the relatively small number of these resistance cells in the initial tumour population could have favoured the homing of NM-TC to the VLA-4 positive cells in mice. As a result, CRIT could have preserved a subclone of MM1.S with low α4 that was present at low frequency in the injected cells. Thus, targeting VLA-4-rich cancer cells selects for the subset of MM1.S cells with low CD49d expression levels and low levels of the activated conformation of VLA-4. A potential approach to achieving complete eradication of MM cells is to identify complementary biomarkers that allow the delivery of TC-loaded NM to all MM1.S cells or through the use of combination therapy that more effectively targets and eliminates both CRIT-responsive MM1.S and MM1.S^CRIT-RES^ cells in vivo.

### CRIT preserves normal hematopoietic stem cells

An important consideration during CRIT is to preserve and sustain the long-term viability of the hematopoietic stem cells and progenitor cell population in the bone marrow. Clonogenic assays of normal bone marrow progenitor cells extracted from mice treated with CRIT did not reveal a significant change in colony-forming units (CFU) compared to the control groups (Fig. 4i). Competitive bone marrow repopulation experiments showed that there was no detrimental effect of CRIT on the primitive hematopoietic stem cell compartment^33^. The 2-month hematopoietic reconstitution of mice transplanted with bone marrow from wild-type vs. CRIT-treated mice was not significantly different, suggesting that there was no obvious reduction in engrafting hematopoietic stem cell population after treatment with CRIT (Fig. 4j, k).

### HSA-TC nanoparticles deliver drug to metastatic breast cancer

We extended the use of CRIT in a metastatic breast cancer model. PyMT-BO1 cell line is a highly aggressive breast cancer cell line derived from transgenic PyMT breast cancer mice^15^. Previous reports have demonstrated that rapidly proliferating tumour cells actively internalize albumin for use as a source of nitrogen and energy, partially accounting for the formulation of drugs in albumin or its nanoparticles for drug delivery to tumours^34–36^. Thus, formulation of hydrophobic drugs, such as TC in albumin, will not only enhance solubilization in an aqueous medium, but also mediate delivery to tumours. We mixed TC (80 mg) in an aqueous solution (16 mL) containing 0.5% human serum albumin (HSA) for 6 h before lyophilizing the entire mixture (see 'Materials and Methods' section). We used a low concentration of HSA in this formulation to prevent potential immunogenic response in mice. The lyophilized product was reconstituted in 0.9% saline immediately before use. Using inductively coupled plasma mass spectrometry, we determined the concentration of TC in the reconstituted sample as 5.6 mg mL^−1^ (Table 2). The corresponding concentration of HSA in the formulation was 8.95 mg mL^−1^, as determined by using a protein assay. Dynamic light scattering (DLS) measurements indicate that the HSA-TC nanoparticles were fairly monodispersed with an average size of 12–15 nm (Supplementary Figure 3). About 1.5% of the particles formed large aggregate clusters of 100 nm, creating a bimodal distribution that skewed the z-average diameter (120 nm) and polydispersity index (0.278). Electron microscopy size measurement correlated with the DLS results, showing mostly monodispersed HSA-TC nanoparticles of 10–15 nm in diameter (Supplementary Figure 4), along with few aggregates of 30–90 nm. The HSA nanoparticles allowed us to load high concentration of the hydrophobic TC per volume of aqueous solution for subsequent delivery to metastatic breast cancer.

### Biodistribution of HSA-TC in metastatic breast cancer model

**Fig. 5:**
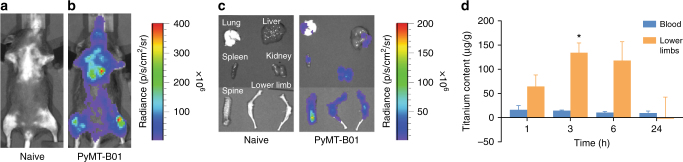
Dissemination of metastatic breast cancer cells and biodistribution of TC in mice. **a** In vivo BLI non-tumour-bearing C57BL/6J mice. **b** In vivo BLI of metastatic breast cancer 10 days post intracardiac injection of PyMT-BO1 GFP/Luc in C57BL/6J mice. **c** Ex vivo BLI of metastatic tumour burden 10 days post tumour initiation. The left panel are tissues obtained from **a** mouse and the right panel are tissues obtained from **b** mouse. **d** Inductively coupled plasma mass spectrometry analysis of Ti content in blood samples and the lower limbs, where tumour burden is high. The Ti content was background-corrected from untreated mice; *P < 0.05. Studies were performed with *n* = 5 mice per each group

Intracardiac injection of PyMT-BO1 cells stably transfected with GFP-firefly luciferase in mice-induced bone metastases, especially to the lower limbs and other major organs (Fig. 5a, b). By day 10, the tumour burden was very high, requiring immediate killing (Supplementary Figure 5). For the biodistribution study, we reconstituted the lyophilized HSA-TC in saline and administered 100 µL of 0.6 mg per 20 g mouse. Both the non-invasive in vivo (Fig. 5b) and the ex vivo (Fig. 5c) BLI analysis showed that the tumour cells disseminated to most major organs, including the vertebrae and lower limbs. Quantitative analysis of the Ti contents in tissue samples from blood and lower limbs by inductively coupled plasma mass spectrophotometry showed the highest accumulation of the metal in the limbs at 3 h post injection of HSA-TC (Fig. 5d). The concentration of Ti in the lower limbs decreased gradually over time (*p* < 0.05). At 24 h, the Ti content from the HSA-TC in the limbs was indistinguishable from the background content. We had to subtract the background Ti from untreated mice to determine the contribution of HSA-TC because most mouse feedstock contains Ti products.

### HSA-TC inhibits growth of metastatic breast cancer in mice

**Fig. 6:**
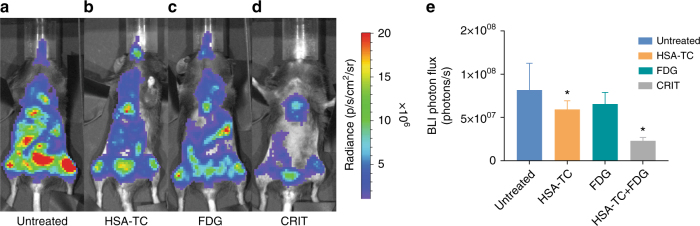
Representative BLI of PyMT-BO1 GFP/Luc metastatic breast cancer cells in C57B6. **a** Untreated C57B6 mouse bearing highly metastatic PyMT-BO1 cancer. Accumulation in the lower limbs were predominant. **b** Mouse treated with 30 mg kg^−^^1^ of HSA-TC nanoparticles. **c** Mouse treated with 800 µCi of ^18^FDG. **d** Mouse treated with a combination of HSA-TC and 800 µCi ^18^FDG. **e** Quantification of whole-body luminescence in CRIT-treated mice compared to untreated, HSA-TC treated or ^18^FDG-treated controls (*P values are 0.038, 0.23 and 0.017 for CRIT, HSA-TC alone and ^18^FDG alone, respectively). BLI and data analysis were performed on day 9 after initiation of PyMT-BO1 metastasis in mice. Studies were performed with *n* = 5 mice per each group

We explored the feasibility of using CRIT to inhibit tumour growth in the highly metastatic PyMT-BO1 GFP/Luc breast cancer model in C57B6 mice. Our biodistribution data indicate that the accumulation of TC in the cancer-homing organs is highest at about 3 h post injection of HSA-TC. ^18^FDG (50–60 µL; 800 µCi per mouse) was administered intraperitoneally at 2 h after the intravenous administration of HSA-TC. We chose intraperitoneal instead of intravenous route for ^18^FDG injection to maintain consistency across the experiments because of the difficulty of finding viable tail veins in the same mouse for multiple injections of both HSA-TC and ^18^FDG. Comparison of different routes of ^18^FDG administration in mice determined that the SUV of ^18^FDG injected intraperitoneally in tumours is optimal at about 1 h post injection, and is similar to intravenous route at closer to this time point^37,38^. We hypothesized that administering the radionuclide at about 2 h after injection of the HSA-TC (100 µL of 0.6 mg per 20 g mouse) will achieve maximum accumulation of both CRIT effectors in tumours after 3 h. Three treatment cycles of HSA-TC and ^18^FDG were administered 2 days apart starting from day 2 after initiating the metastatic disease when the tumours are observable by BLI (Supplementary Figure 5). Groups with no treatment, treatment with HSA-TC alone and ^18^FDG alone served as controls. Whole-body luciferase activity from day 2 to day 9 were analysed for tumour cell proliferation. Compared to the untreated mice (Fig. 6a), a small decrease in BLI signal was observed in the HSA-TC (Fig. 6b) and ^18^FDG (Fig. 6c)-treated mice compared to the untreated cohort. However, the pattern of tumour growth in all the three control groups was similar. In contrast, the CRIT group showed significant tumour stasis, with a few focused cluster of tumour cells that did not respond to the therapy (Fig. 6d, e). The slow growth and focal nature of the CRIT-resistant cells suggests that this therapeutic method can transform metastatic cancer into a surgical disease. Whereas PyMT-BO1 model is an excellent model for the rapid evaluation of drugs or treatment methods, all the animals eventually died or were killed between day 9 and 12 due to the aggressiveness of this model. The fast death cycle prevents longitudinal evaluation of each animal, which is needed to obtain reliable survival plots. Future studies will explore different models and establish the mechanism of therapy resistance in this cancer cell line.

In summary, we have successfully demonstrated the application of PT for treating disseminated malignancies using VLA-4-targeted NM and HSA-TC nanoparticles, activated by radiopharmaceuticals. Integral to this strategy is the availability of a wide range of radionuclides for clinical PET imaging and preclinical Cerenkov luminescence imaging to further monitor and guide treatment response^39,40^. We demonstrated a strategy to rescue abandoned light-sensitive drugs with poor therapeutic outcomes such as TC and some FDA-approved drugs with inherent photoactivity into precision phototherapeutics. In addition, clinical biochemistry parameters and histopathologic assessment of vital organs in the treated and untreated controls were similar (Supplementary Figure 6 and Supplementary Figure 7). The brain, heart, liver and kidneys were of particular interest because ^18^FDG naturally accumulates in these organs because of their high glucose utilization and elimination pathways. The absence of off-target toxicity to normal hematopoietic stem cells may favour the translation of this approach in the clinic as either a standalone therapy or as a combination with other therapies, including chemotherapy, where the suppression of the bone marrow and the risk of pancytopenia may not be anticipated to be greater than those patients receiving chemotherapy without CRIT. Our results suggest that the sequential administration of the NM-TC and radionuclide minimizes the association of both therapeutic components in vital organs. These findings expand the potential use of PT for treating previously PT-inaccessible metastatic, infectious and cardiovascular diseases.

## Methods

### Synthesis and characterization of VLA-4-targeted titanocene micelles

The VLA-4 ligand, LLP2A (Supplementary Figure 2a), was prepared on solid support using standard fluorenylmethyloxycarbonyl (Fmoc) peptide synthesis as reported previously^41^. Starting with Rink Amide resin, serial Fmoc deprotection cycles were achieved with 20% piperidine in dimethylformamide (DMF) and coupling of amino acids was performed with hydroxy-benzotriazole (HOBt) and 1,3-diisopropylcarbodiimide (DIC) in DMF at 25 °C. The crude product was cleaved from the resin with a mixture of 95% trifluoroacetic acid (TFA): 2.5% water: 2.5% triisopropylsilane and precipitated with cold diethyl ether. The product was purified by RP-HPLC and characterized by analytical HPLC and ES-MS: calculated mass for LLP2A is 1084.27 Da; observed mass is 1085 Da (Supplementary Figure 2b). To incorporate LLP2A into nanomicelles, LLP2A was dissolved in ethanol and mixed with 2-iminothiolane in methanol. After reacting for 2 h at 25 °C, polyethylene glycol_2000_-phosphatidylethanolamine (PEG-PE) was added to the mixture and incubated for another 2 h. The product was purified using 3000 Da MWCO dialysis tubing to dialyse off the free LLP2A, and lyophilized to give a white solid (LLP2A-PEG-PE). The product was characterized by analytical HPLC and ES-MS: average calculated mass for LLP2A-PEG-PE is about 4100 Da; observed mass is about 4065 Da (Supplementary Figure 2c). The phospholipid/polysorbate 80 micelles were prepared as a microfluidized suspension comprising 20% polysorbate Tween 80 (v/v), a 2.0% (w/v) surfactant commixture and 1.7% (w/v) glycerine in filtered MilliQ Nanopure water. The surfactant co-mixture were 0 or 2 mole% TC and 0 or 0.15 mole% of LLP2A-PEG-PE, with the remainder as phosphatidylcholine (>98% purity, NOF America). The surfactant components were dried from organic solvent into a film, resuspended in nanopure water, and combined with polysorbate 80 and glycerine mixtures, followed by sonication at 4 °C for 3 min and then microfluidized (LV1, Microfluidics, Inc) at 20,000 psi for five passes. The micelles were filtered with a 0.2 µm filter into sterile serum vials, preserved under inert gas, capped and crimp-sealed before storage at 4 °C for subsequent use. The concentration of TC in TC nanomicelles was determined by inductively coupled plasma optical emission spectrometry (PerkinElmer Optima 5300). For each measurement, 20 µL reconstituted TC nanomicelle (*n* = 3) was digested with concentrated nitric acid in Mars 6 Microwave Digestion System (CEM Corporation). Microwave power was ramped up to 200 °C for 20 min followed by a hold for 20 min at the same temperature. All external calibration standards of 0.01, 0.1, 1.0, 10, 50, 100 and 200 µg L^−1^ were within 10% of expected concentration. Relative standard deviation measurements were less than 5% for all standards and the samples.

Dynamic light scattering measurements were acquired with a Zeta Plus Zeta Potential Analyzer (Brookhaven Instruments Corporation, Holtsville, NY) equipped with a 633 nm laser. Three measurements were conducted in deionized water for each sample with at least 10 runs and each run lasting 10 s. All sizes reported were based on intensity average. For negative staining and electron microscopy analysis of NM, samples were allowed to absorb onto Formvar/carbon-coated copper grids for 10 min. Grids were washed two times for 1 min each in dH_2_O and stained with 1% aqueous uranyl acetate (Ted Pella Inc., Redding CA) for 1 min. Excess liquid was gently wicked off and grids were allowed to air dry. Samples were viewed on a JEOL 1200EX transmission electron microscope (JEOL USA, Peabody, MA) equipped with an AMT 8-megapixel digital camera (Advanced Microscopy Techniques, Woburn, MA).

### Synthesis and characterization of HSA-TC

TC (80 mg) was added in an aqueous solution (16 mL) containing 0.5% HSA. The mixture was shaken moderately at 560 oscillation per min in IKA KS 130 basic plate shaker for 6 h at room temperature, followed by lyophilization in Thermo Fischer SAVANT RVT5105 refrigerated vapour trap lyophilizer to obtain the HSA-TC as dry powder. The lyophilized product was reconstructed in 0.9% saline immediately before use. The concentration of the TC in the reconstituted injection was 5.6 mg mL^−1^ (characterized by inductively coupled plasma optical emission spectrometry and that of HSA in the reconstituted solution was 8.95 mg mL^−1^ (determined by Bio-Rad Quick Start Bradford Protein Assay kit). The size and dispersity of the HSA-TC was confirmed by DLS (Malvern Zetasizer nano Series) and electron microscopy (JEOL TEM-1400 electron microscope).

### Cell culture

All cell lines underwent STR profiling and tested for mycoplasma contamination. MM1.S-Luc cells were cultured in RPMI1640 medium containing 10% heat-inactivated FBS and 2 mercaptoethanol (50 μM final) and 1× of all of the following: penicillin/streptomycin (100 μg mL^−1^ final), sodium pyruvate (1 mM final), non-essential amino acids, HEPES (10 mM final) and l-glutamine. A similar condition was used to culture PyMT-BO1 cells in DMEM media plus 10% FBS.

### Tumour models in mice

All animal studies were conducted in compliance with the guidelines established by the Animal Studies Committee at Washington University in St. Louis, Missouri. Fox Chase SCID Beige mice (4-week, female) were purchased from Charles River laboratories for developing the disseminated MM model. C57BL/6J mice (6-week, female) were purchased from the Jackson Laboratory for developing the metastatic breast cancer model. MM1.S-Luc cells (1 × 10^6^ in 100 µL per mouse) were injected intravenously for the MM model and PyMT-BO1-GFP-Luc cells (1 × 10^5^ in 50 µL per mouse) were injected in the left ventricular chamber for the breast cancer model. BLI was used to monitor cell viability and tumour burden.

### Bioluminescence imaging

Ex vivo and in vivo bioluminescence imaging of MM1.S-Luc in SCID mice and PyMT-BO1 GFP/Luc in C57BL/6J was performed on an IVIS Lumina (PerkinElmer, Waltham, MA; Living Image 4.3, 5 min to 1 s exposure, bin2–8, FOV12.5 cm, f/stop1, open filter). Image analysis was performed using Living Image 2.6 software. For in vitro imaging, optimal bioluminescence for subsequent in vivo studies was determined by plating 1 × 10^5^ cells in a black 24-well plate. Fixed regions of interest (ROIs) were drawn on each well and images captured after an exposure time of 10 s. A radiance >1 × 10^8^ photons s^−1^ cm^−2^ sr^−1^ was considered as the threshold. For in vivo imaging, mice were injected intraperitoneally with D-luciferin (150 mg kg^−1^ in PBS; Gold Biotechnology, St. Louis, MO) and imaged after anaesthetizing with isoflurane (2% vaporized in O_2_). Total photon flux (photons s^−1^ cm^−2^ sr^−1^) was measured from fixed ROIs over the entire mouse.

### Pharmacokinetics of NM-TC in rats

For pharmacokinetic analysis, the NM at a dose of 25 μL kg^−1^ was administered intravenously to rats (*n* = 5) and serial blood samples were drawn at 1, 5, 15, 30, 60, 90, 120 and 1440 min for metal analysis. The PK in rats is done to allow enough sample for serial measures in the same animal. Otherwise, blood samples must be pooled, masking inter-specimen variability. Briefly, animals are weighed, anesthetized and shaved on the ventral side of the neck. The surgical area is cleaned and wiped free of hair before surgery. Following stabilization, the incision site is anesthetized, and an incision made just off midline to the trachea through the skin. The carotid artery is exposed by blunt dissection and snared with two ligatures, one proximal one distal, for introducing the catheter. The wound is wetted periodically with lidocaine (2%)/bupivicaine (2.5%) and saline to maintain analgesia. The distal tie is tightened to ligate the artery, and the proximal tie is made into a Potter’s knot. A silastic catheter is inserted through an arteriotomy and secured in place with the ligatures. The catheter is then flushed with heparinized saline. Blood is sampled via a carotid catheter. Blood samples were ~150 μL and flushed with equivolume of saline. The blood samples were digested by microwave digestion in HNO_3_/H_2_O_2_ under pressure and the concentration of TC (mg mL^−1^) was determined by inductively coupled plasma optical emission spectrometry and reported as percentage of injected dose per gram of tissue (ID g^−1^).

### Biodistribution of NM-TC in mice

The NM-TC at a dose of 50 μL per mouse were administered intravenously in MM1.S (*n* = 5) tumour-bearing mice. A one-time dose was administered 30 days post cell injection of MM1.S cells. Tf-TC and MKT4 were prepared as described in the literature^18^. Briefly, MKT4 was prepared by adding fivefold molar excess of mannitol (500 mg) to TC (100 mg) and a 19-fold molar excess of sodium chloride (450 mg) in 50 mL water. The solution was mixed in a shaker at 25 °C for 4 h before lyophilizing. For intravenous injections, MKT4 was reconstituted in saline at a dose of 0.25 mg kg^−1^. To prepare Tf-TC, fivefold molar excess of TC was added to human apo-Tf and incubated in a shaker for 2 h at 25 °C. A working stock of TC was initially prepared in DMSO due to low solubility of TC in water and aqueous buffers. The mixture was then dialysed overnight against DPBS using a 3000 Da molecular weight cutoff (MWCO) Slide-A-Lyzer MINI Dialysis Devices to remove excess TC. MKT4 and Tf-TC were administered intravenously at a dose of 0.25 mg kg^−1^ (normalized to the TC content in NM) in MM1.s-SCID mice (*n* = 5). The mice were then killed 90 min post injection and the organs were dissected. The organs and blood were processed by microwave digestion in HNO_3_/H_2_O_2_ under pressure and the TC measurements were measured in mg mL^−1^ by inductively coupled plasma optical emission spectrometry and adjusted to %ID g^−1^ tissue.

### Biodistribution of HSA-TC in mice

A similar procedure described above was used in the HSA-TC study. HSA-TC (50 µL per mice, 30 mg kg^−1^) was injected intravenously in C57B6 mice on day 10 post intracardiac injection of PyMT-BO1 GFP/Luc breast cancer cells. In addition to the untreated group, each group of five mice were killed at 1, 3, 6 and 24 h post injection after BLI of the living animal. The organs and blood were harvested and imaged ex vivo using BLI to estimate the tumour distribution. To determine uptake of the Ti distribution, blood and lower limbs at the different time points were were digested by microwave in HNO_3_/H_2_O_2_ under pressure and analysed by inductively coupled plasma mass spectrometry. The Ti content in each tissue were measured in mg mL^−1^ after subtraction of background Ti from the untreated cohorts.

### ^18^FDG-PET imaging

^18^FDG-PET imaging was performed on MM mice 30 days post cell injection (*n* = 4) along with naive mice (*n* = 4). The mice were fasted for 6 h before each scan. After anesthetizing the mice with 1.5–2% isoflurane and oxygen, 0.19 mCi (7.03 MBq) 0.1 mL^−1^ of FDG was administered intravenously. A 10-min transition scan was performed just before the 10 min emission at 1 h post injection using a MicroPET-Inveon MultiModality scanner (Siemens Preclinical Solutions, Erlangen, Germany). The animals were placed on the microCT^®^ in the same position to obtain anatomical imaging and co-registered to the microPET^®^ image. The data were analysed using Inveon Research Workstation software, by manually drawing three-dimensional ROI from PET images using CT anatomical guidelines. The activity associated with tumour was measured and maximum SUVs were calculated using SUV = ([mCi mL^−1^] x [animal weight (g)]/[injected dose (mCi)]).

### In vivo CRIT in disseminated MM1.S-luc/SCID mouse model

After MM1.S cell injection, considered day 1, mice were imaged weekly using BLI for 7–9 weeks. Treatment was initiated on day 5. Mice were administered 50 μL of NM-TC (0.25 mg kg^−1^ TC) intravenously followed by 29.6 MBq 0.1 mL^−1^ of ^18^FDG 90 min later, also administered intravenously (*n* = 15). Control mice (*n* = 5 per group) were administered with NM-TC or ^18^FDG alone. A total of four treatment cycles at an interval of 1 week were given per animal, where a cycle refers to an administration of NM-TC and ^18^FDG. BLI and survival of these groups of mice were tracked along with untreated controls (*n* = 10) on a weekly basis. Food was withheld from mice for 6 h before administering ^18^FDG and kept in a dark, lead-shielded room post injection. The weight and any physical signs of distress were also monitored closely. The mice were killed by cervical dislocation after anaesthesia with 5% isoflurane when there was a loss of >20% total body weight. For rechallenging studies on resistant MM1 cells (MM1^CRIT-RES^), the bone marrow (BM) was harvested from treated mice when whole-body radiance exceeded 5 × 10^4^ photons s^−1^ cm^−2^ sr^−1^. BM was harvested by flushing the femoral shaft with PBS and reinjecting the cells into a fresh set of Fox Chase SCID Beige mice. The treatment schedule followed was similar to MM1.S described above. BLI was used to track and compare treatment response in a CRIT group (*n* = 10) and an untreated group (*n* = 10).

### In vivo CRIT in metastatic breast cancer PyMT-BO1 model

A similar procedure described above was followed in the HSA-TC CRIT study. On day 2 post intracardiac injection of PyMT-BO1-GFP-Luc cells in C57BL/6J mice, BLI was used to confirm tumour viability and engraftment. The mice were stratified into the following groups: untreated control, HSA-TC only, ^18^FDG only and combination of HSA-TC and ^18^FDG cohorts. Each group has five mice weighing between 18 and 20 g. All mice were fasted 3 h before commencing the treatment on day 2. HSA-TC (100 µL per mice, 30 mg kg^−1^ was injected intravenously in the HSA-TC and HSA-TC + ^18^FDG groups. This was followed by intraperitoneal (i.p.) injection of the ^18^FDG (800 µCi in 50 µL saline containing 0.02% ethanol) in the ^18^FDG-only group and the HSA-TC + ^18^F FDG group after 2 h from the drug injection. This treatment regimen was performed every 2 day for 3 times and BLI images were captured first on each occasion before the therapy. Tumour response was quantified using both whole body and then lower limb signals from BLI.

### RNA analysis

Bone marrow was flushed from femurs using cold PBS and single cell suspensions were prepared by filtering through a 40 μm nylon strainer. Red blood cells were lysed using Red Blood Cell Lysing Buffer (Sigma-Aldrich) and washed with cold PBS. MM1^CRIT-RES^ cells were sorted by fluorescent-activated cell sorting for GFP. RNA was extracted from MM1^CRIT-RES^ cells using the Qiagen RNAeasy mini kit according to the manufacturer’s instructions. cDNA was synthesized using qRT-PCR was performed on the Applied Biosystems StepOnePlus Real-Time System (Thermo Fisher) using pre-designed TaqMan^®^ Gene Expression Assays (Life Technologies; 18S RNA Hs99999901 and glut1 Hs00892681) according to the manufacturer’s instructions. The ΔΔCT method was used to calculate changes in fold expression and results were analysed with GraphPad Prism 6.

### Flow cytometry

Red blood cells in the flushed BM were lysed using Red Blood Cell Lysing Buffer (Sigma-Aldrich) and washed with cold PBS. Cell subsets were evaluated by flow cytometry by re-suspending the cells in staining buffer (PBS supplemented with 0.5% bovine serum albumin and 2 mM EDTA) and incubating for 30 min at 4 °C with pre-titrated saturating dilutions of the following fluorochrome-labelled monoclonal antibodies: CD49d-PE/Dazzle594 (clone 9F10; Biolegend), CD29-APC (clone TS2/16; Biolegend), human CD45-Alexa Fluor 700 (clone HI30; Biolegend), CD138-BV421 (clone B-B4; Biolegend) and murine CD45-BV510 (clone 30-F11; BD Biosciences). To measure LLP2A binding, cells were washed and resuspended in Hanks Balanced Salt Solution (HBSS; Lonza) containing Ca^2+^ (1.26 mM), Mg^2+^ (0.81 mM) and 0.1% bovine serum albumin (Sigma-Aldrich). Aliquots of cells were left untreated or treated with 10 nM BIO5192 (Tocris) at 4 °C for 15 min. Samples were then incubated with or without LLP2A-Cy5 (0.41 µg µL^−1^) for 30 min at 4 °C and washed twice with HBSS.1 Dead cells were excluded from these assays by staining with 2 mg mL^−1^ 7-amino-actinomycin D (Molecular Probes, Eugene, OR) for 5 min before analysis. Samples were analysed on a Beckman Coulter Gallios flow cytometer and data were analysed using FlowJo software (TreeStar, Ashland, OR). Statistical comparisons of flow cytometry data were performed using an unpaired two-tailed Student's *t* test (GraphPad Prism). *P* values ≤0.05 were considered significant.

### Colony-forming unit assay

For enumeration of blood colony-forming cells (CFU), aliquots of bone marrow cells were incubated in duplicate in commercially available growth factor-supplemented methylcellulose medium for mouse CFU-C (Stem Cell Technologies, Vancouver, BC) as described. Blood CFU were enumerated after 6–8 days of culture.

### Competitive bone marrow transplant

C57BL/6 mice (B6.CD45.2) were treated with either CRIT (*n* = 5) or PBS control (*n* = 5). BM was harvested on day 40 by flushing the femoral shaft with PBS. Bone marrow from treated mice was mixed with congenic BM (B6.CD45.1/2) at a ratio of 1:1 before infusion of 1 × 10^6^ total BM cells into lethally irradiated (TBI 1100 cGy) B6.CD45.1 recipients via lateral tail vein injection. Peripheral blood was analysed by flow cytometry at week 8+ to quantify engraftment of donor BM. Percentage of cells derived from treated donor BM (CD45.2) was calculated as a percentage of total donor BM (CD45.2 + CD45.1/2).

### Statistical analysis

Statistical significance was measured by Student’s *t* test using GraphPad Prism software (GraphPad, San Diego, CA). Kaplan–Meier survival curves were plotted using GraphPad Prism software. Unless noted otherwise, all values are means and error bars are standard deviations. For animal studies, sample size estimates depend on the effect size (mean difference between untreated and treatment groups/SD) of the outcome. For effect size of 2.1 and using a two-sided *t* test, typically five per group were needed with 80% power to detect a significant difference at a type I error rate of 0.05. There were no exclusion criteria, except for the breast cancer study where the use of female mice was needed to recapitulate the histopathology of female breast cancer. There was no blinding of investigators in this research, including animal study.

### Data availability

The data that support the findings of this study are available from the corresponding author on reasonable request.

## Electronic supplementary material

Supplementary Information
