# Supplementary material for: Radionuclides transform chemotherapeutics into phototherapeutics for precise treatment of disseminated cancer
Source: Nat Commun. 2018 Jan 18;9:275. doi: 10.1038/s41467-017-02758-9 (PMC5773683; doi:10.1038/s41467-017-02758-9)
Supplement: Supplementary file 1 — Supplementary Information [file 41467_2017_2758_MOESM1_ESM.pdf]

SUPPLEMENTARY INFORMATION

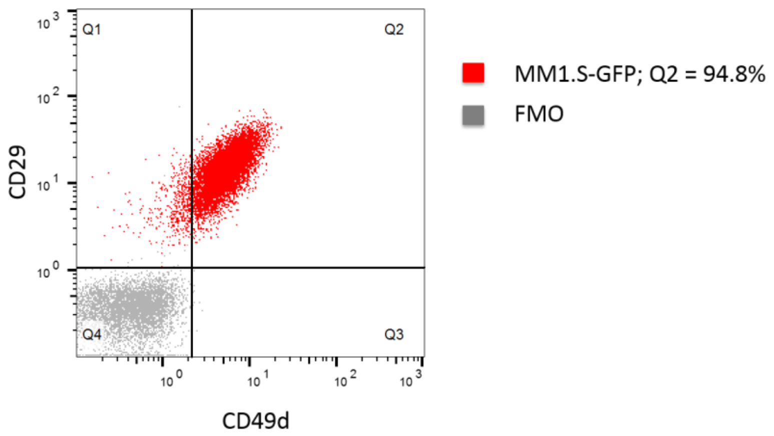

Supplementary Figure 1. VLA4 (CD29^{+}CD49d^{+}) expression on multiple myeloma cell lines MM1.S. The human cell line MM1.S were stained with CD49d-PE/Dazzle594 (clone 9F10; Biolegend), CD29-APC (clone TS2/16; Biolegend) (94.8% VLA4^{+}). Dead cells were excluded from these assays by staining with 2 mg/ml 7-amino-actinomycin D (Molecular Probes, Eugene, OR) for 5 min before analysis. Samples were analyzed on a Beckman Coulter Gallios flow cytometer and data were analyzed using FlowJo software (TreeStar, Ashland, OR).

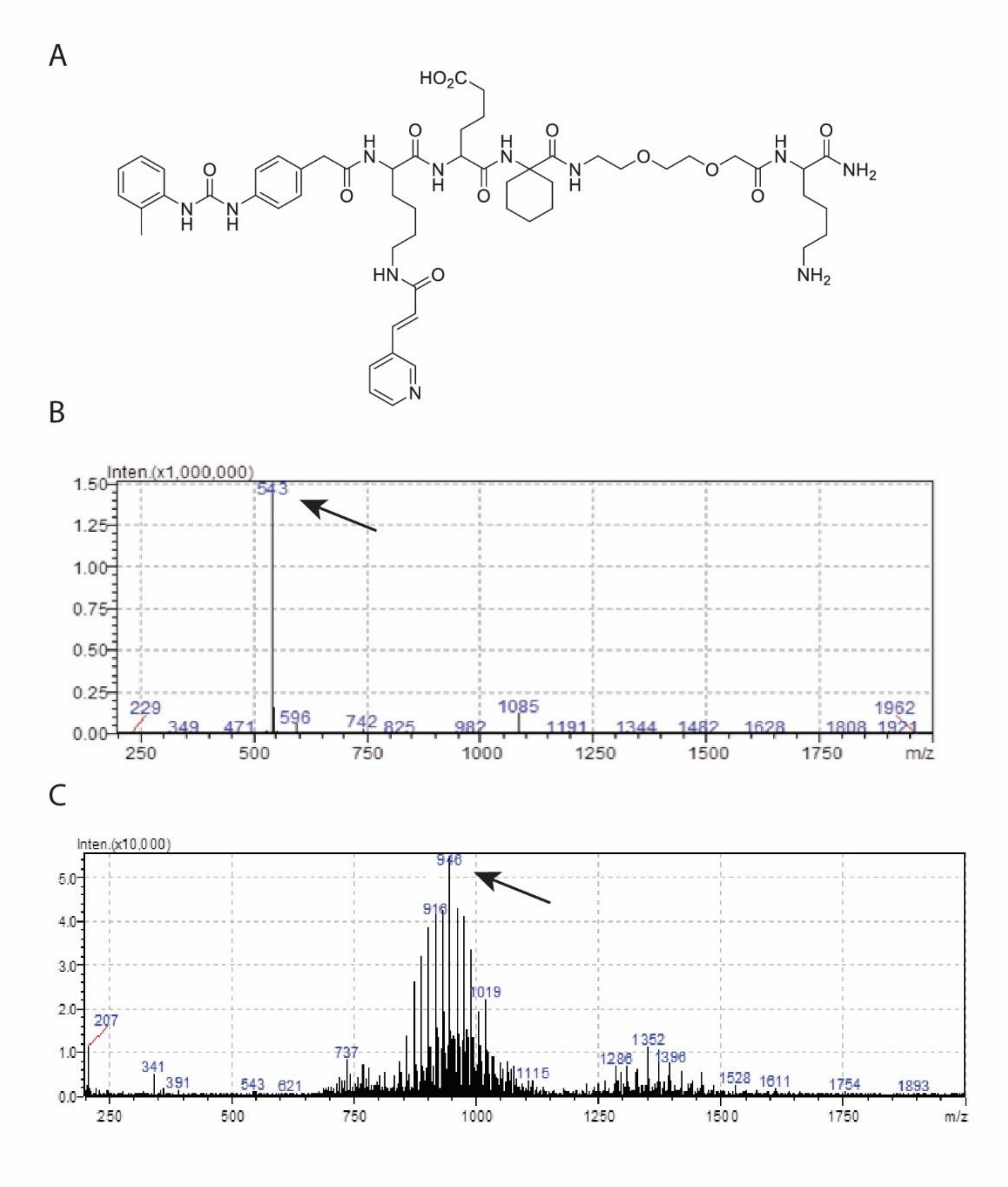

Supplementary Figure 2. A. Structure of LLP2A peptidomimetic. B. Mass spectrometry data of LLP2A (M/2). C. Mass spectrometry data of LLP2A-PEG-PE conjugates (M+4/4).

A B C

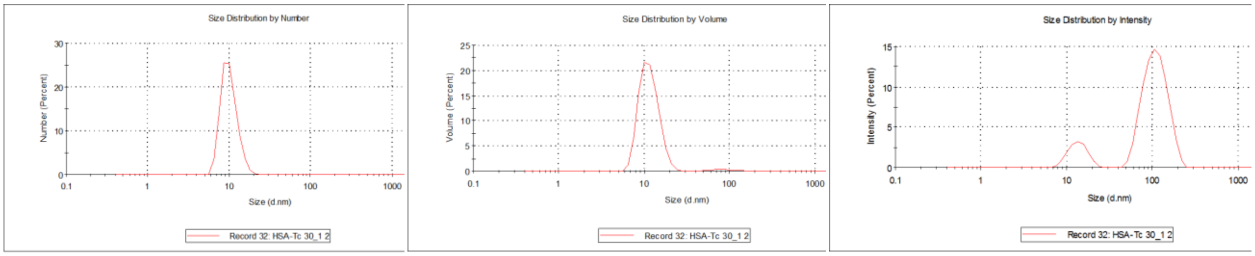

Peaks	Average Size (d/nm)	Number %	St. Dev (d/ nm)
Peak 1	10.14	100	2.371
Peak 2	0.00	0.00	0.00

Peaks	Average Size (d/nm)	Volume %	St. Dev (d/nm)
Peak 1	11.69	98.2	3.061
Peak 2	92.83	1.8	33.36

Peaks	Average Size (d/nm)	Intensity %	St. Dev (d/nm)
Peak 1	13.79	14.8	3.338
Peak 2	112.0	85.2	34.83

Supplementary Figure 3. Hydrodynamic size characterization of the HSA-TC formulations showing particle size analysis by (A) number, (B) volume, and (C) intensity calculations. The intensity-based analysis showed a bimodal distribution, resulting in a skewed z-average diameter of 120 nm and polydispersity index of 0.278.

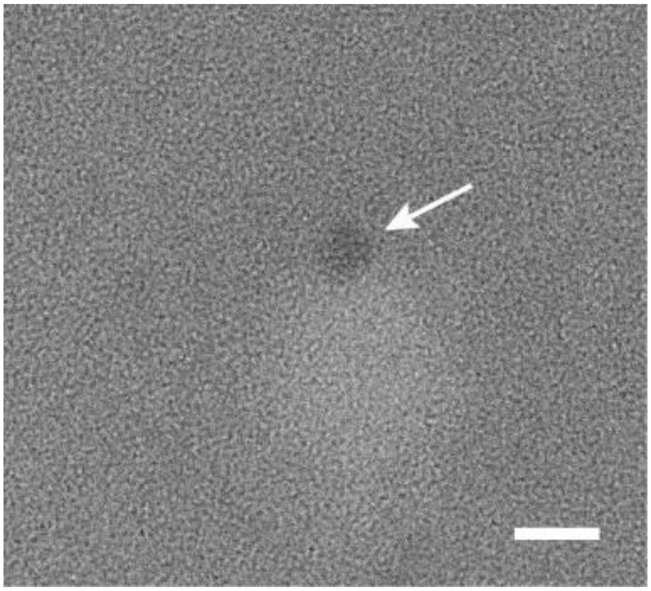

Supplementary Figure 4. A representative TEM image of HSA-TC nanoparticles. Scale bar, 20nm.

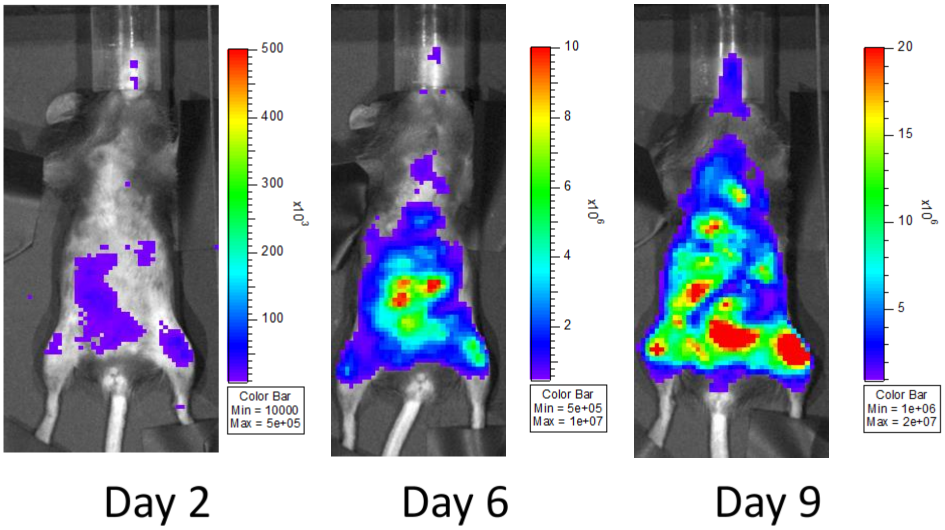

Supplementary Figure 5. In vivo tumor progression in untreated mice after intra-cardiac injection of PyMT BO1 GFP-Luc cells in 6 weeks old C57BL/6J mice.

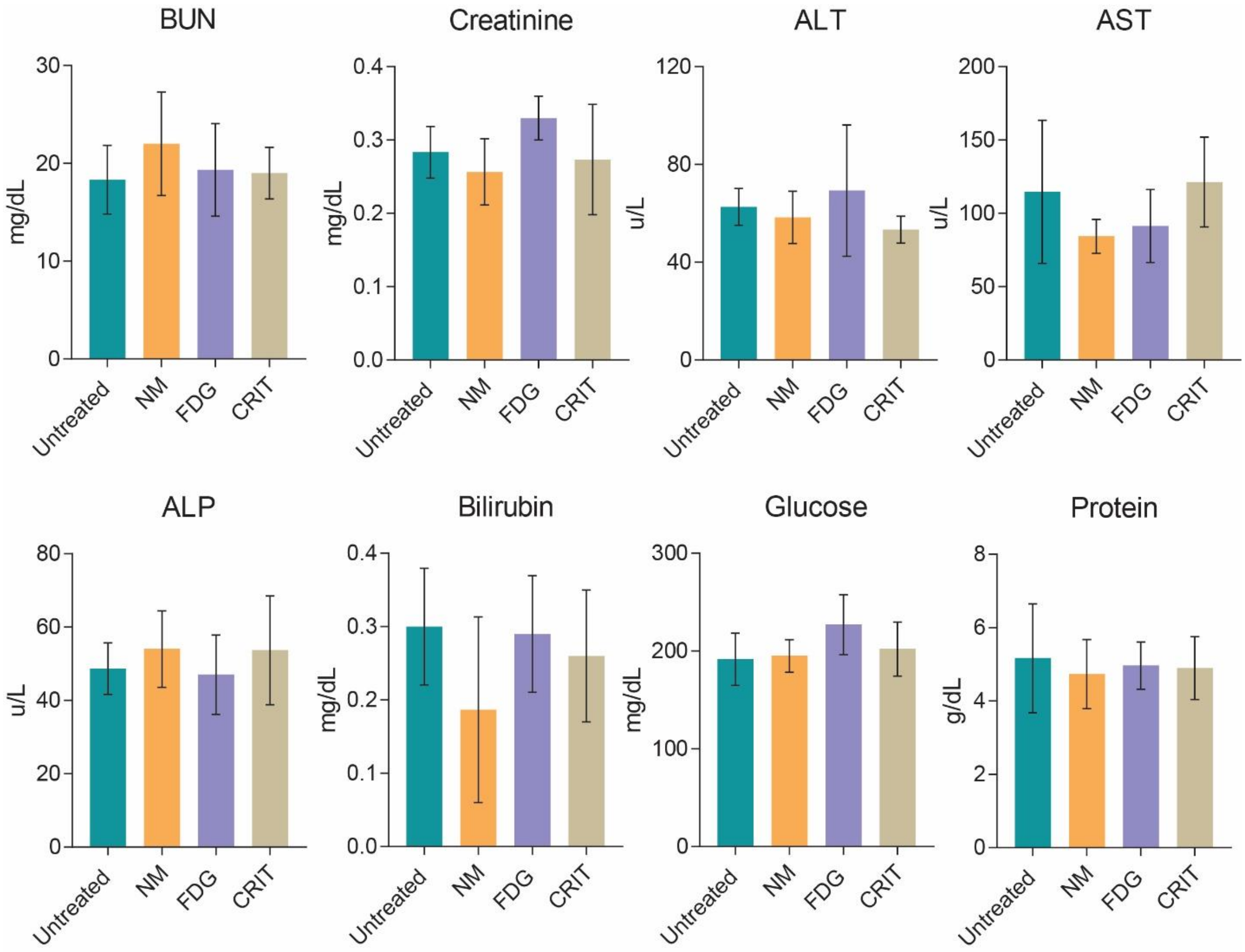

Supplementary Figure 6. Clinical biochemistry parameters were evaluated to determine toxicity of CRIT compared to untreated and single agent controls. Blood was drawn for analysis after full treatment regimen. There was no statistically significant changes observed in any parameter for any of the treated groups when compared to untreated controls. Values are means ± s.e.m.

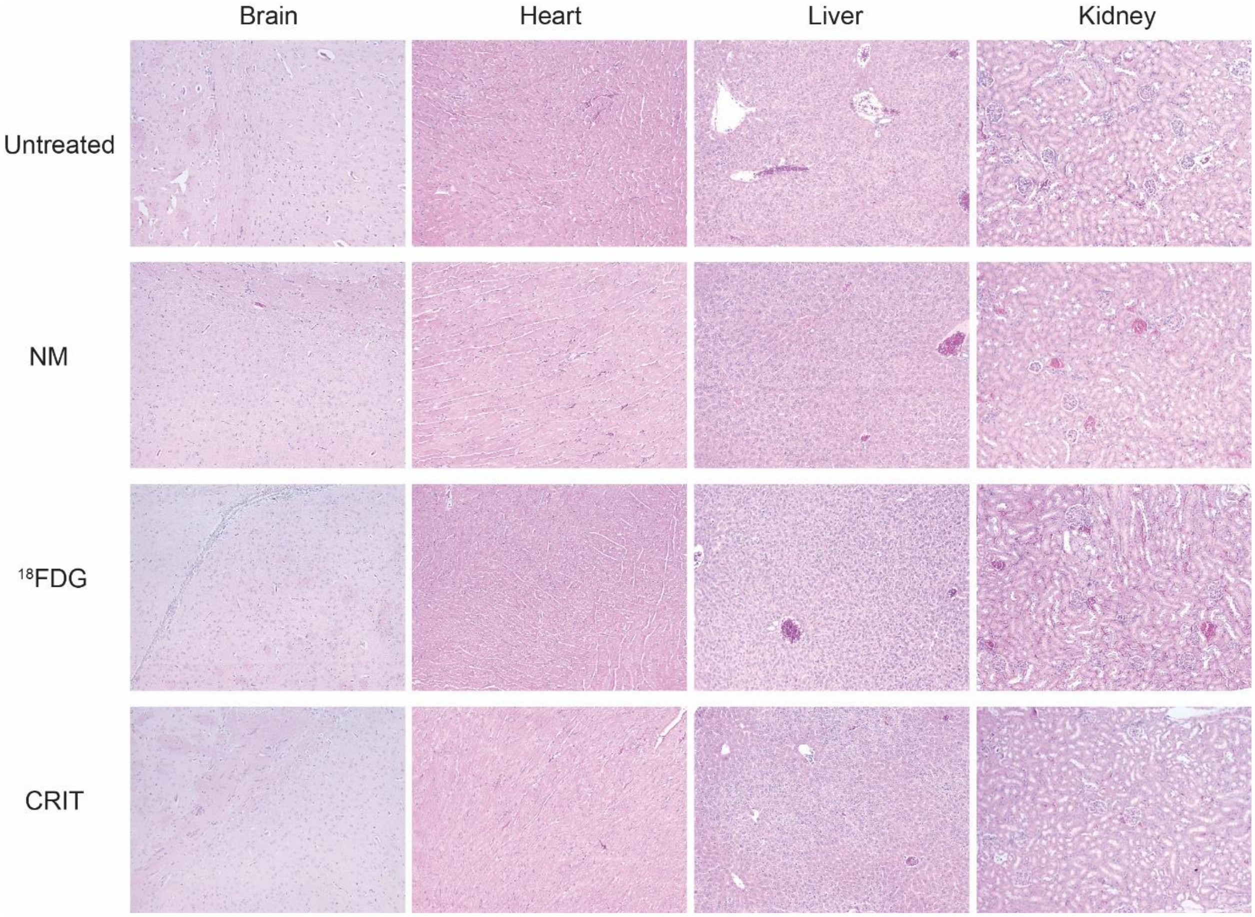

Supplementary Figure 7. Histologic images of tissues at 10X magnification. Comparison of H&E stained brain, heart, liver and kidney sections between different treatment groups did not show significant lesions in these organs, indicating the absence of systemic toxicity from CRIT.
